# Isolated Pulmonary Hydatid Cyst: A Rare Presentation in a Young Maasai Boy from Northern Tanzania

**DOI:** 10.1155/2019/5024724

**Published:** 2019-10-01

**Authors:** Jay Lodhia, Ayesiga Herman, Rune Philemon, Adnan Sadiq, Deborah Mchaile, Kondo Chilonga

**Affiliations:** ^1^Department of General Surgery, Kilimanjaro Christian Medical Center, PO Box 3010, Moshi, Tanzania; ^2^Kilimanjaro Christian Medical University College, PO Box 2240, Moshi, Tanzania; ^3^Department of Pediatrics, Kilimanjaro Christian Medical Center, PO Box 3010, Moshi, Tanzania; ^4^Department of Radiology, Kilimanjaro Christian Medical Center, PO Box 3010, Moshi, Tanzania

## Abstract

**Introduction:**

Hydatidosis is a parasitic manifestation caused by *Echinococcus granulosus.* It is characterized by cystic lesions in the liver and lungs. Diagnosis is based on typical history and radiological measures.

**Case presentation:**

A four-year-old boy presented with a one-year history of dry cough and difficulty in breathing which was of gradual progression. Computed tomography of the chest revealed a large 11.7 cm × 8.6 cm × 11.0 cm cyst in the right hemithorax. The patient underwent thoracotomy and recovered well post procedure.

**Conclusion:**

This case report highlights that large hydatid cysts can be surgically removed with good outcome and the importance of realizing that the disease is a burden to the public health and is much neglected.

## 1. Introduction

Hydatidosis or hydatid disease is a parasitic infection caused by the tapeworm *Echinococcus granulosus* [1, 2]. It is characterized by cystic lesions mainly in the liver [1, 3]. The pathogenesis is due to infestation of a human host by *E. granulosus* following accidental ingestion of dog waste products containing eggs [1]. We report of a case with a pulmonary hydatid cyst in a four-year-old boy.

## 2. Case Presentation

A four-year-old Maasai boy who was accompanied by his elder brother presented to the hospital with a one-year progressive history of dry cough and difficulty in breathing to the extent of compromising the child's physical activity accompanied by intermittent fever. There was no history of tuberculosis contact or trauma but a positive history of living with cattle and dogs. The patient received multiple courses of antibiotics and herbal medication with no relief.

**Figure 1:**
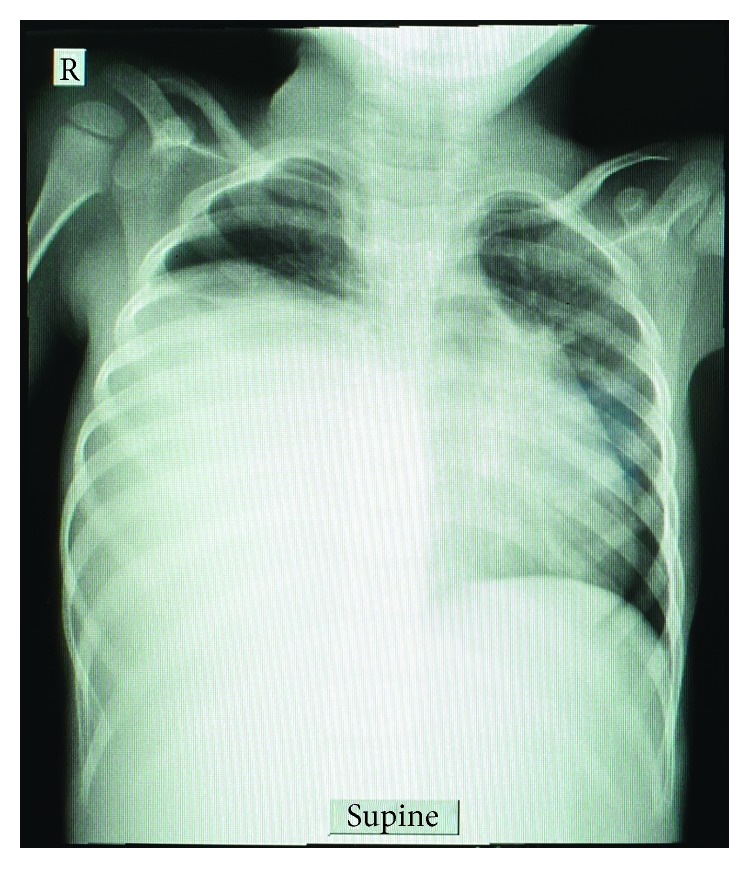
Chest X-ray showing opacification of the right hemithorax.

**Figure 2:**
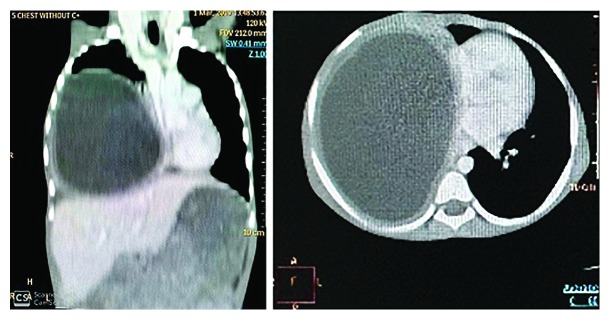
CT scan showing cyst in the right hemithorax.

During admission, the child had a baseline plain chest X-ray done which revealed 80% homogenous opacification of the right hemithorax (Figure 1). For further clarification, chest computed tomography (CT) scan was done which demonstrated a large thick walled cystic lesion in the right hemithorax measuring approximately 11.7 cm × 8.6 cm × 11.0 cm. Fluid in the cyst appeared clear with no solid components, septations, or floating membranes. The right middle and lower lobes were completely collapsed. There was a mediastinal shift towards the left, but the left lung appeared normal. It was concluded that the features were suggestive of a hydatid cyst of the right hemithorax (Figure 2). With this radiologic diagnosis, albendazole was initiated and the patient was prepared for surgery.

**Figure 3:**
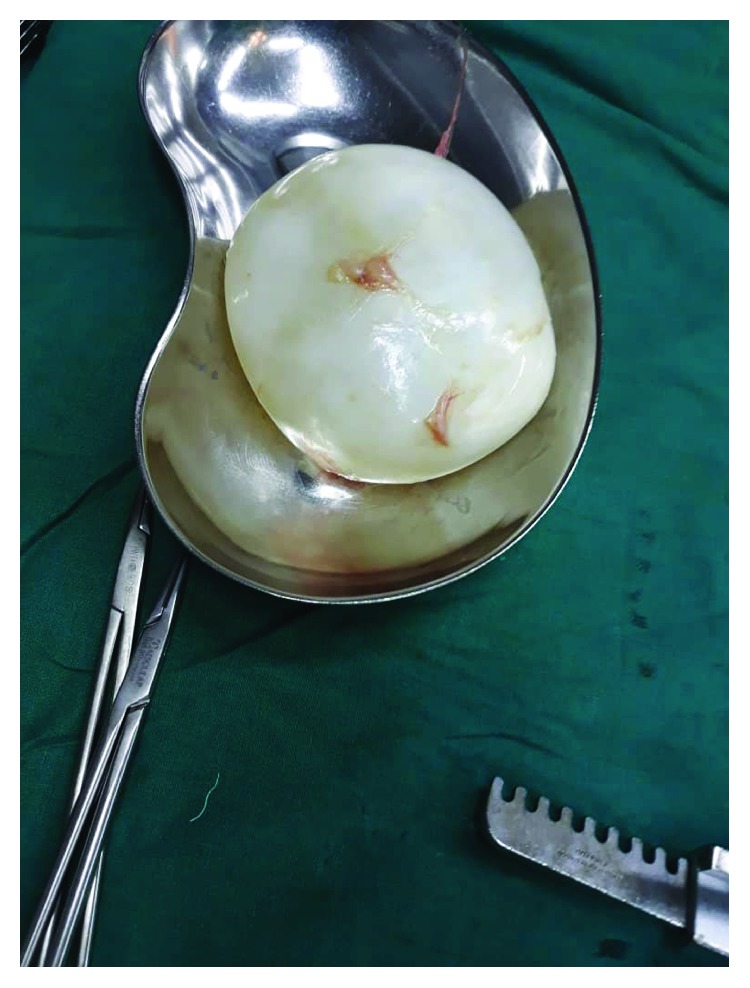
Hydatid cyst.

**Figure 4:**
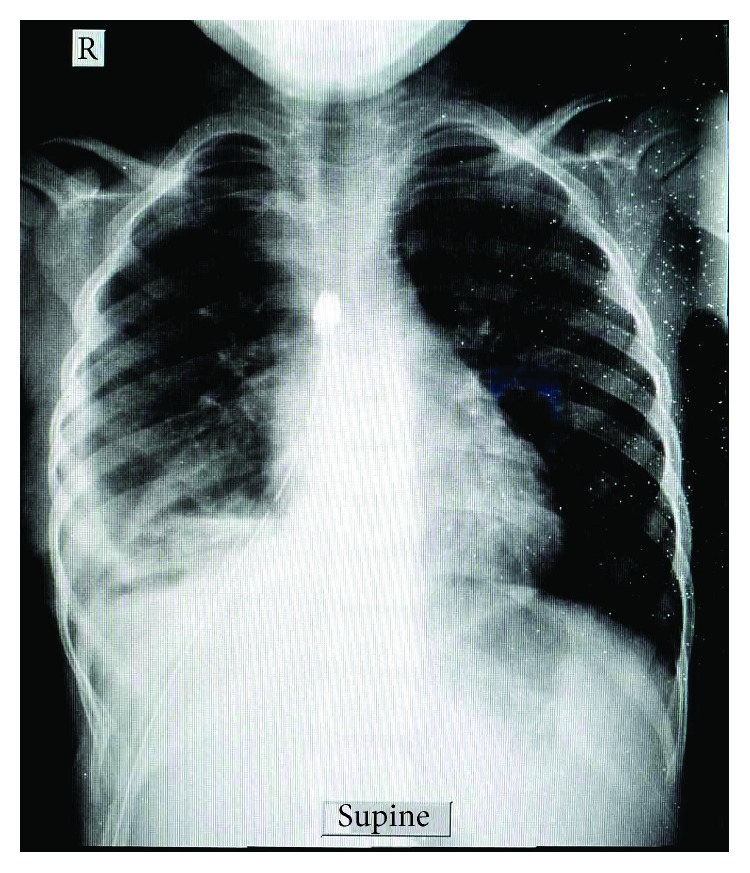
Postoperative chest X-ray with the right thoracostomy tube.

With the consent from the guardian, thoracotomy with right lower lobectomy was done. Intraoperatively, a cyst of about 20 cm in diameter in the lower lobe of the right lung with some fibrin attachment to the right hemidiaphragm was found (Figure 3). The whole cyst was removed with no spillage and a draining tube thoracostomy with underwater seal was placed (Figure 4). The postoperative course was uneventful. The tube thoracostomy drain was removed on day 11 and the patient was discharged on the 12^th^ day.

## 3. Discussion

Hydatidosis is a parasitic infection caused by *Echinoccocus granulosus*. It is endemic in sub-Saharan African countries [4]. Different strains of *E. granulosus* have been identified based on their specific intermediate hosts (e.g., sheep, buffalo, horse, cattle, pigs, camels), and different species of *Echinoccocus* cause different diseases in humans, i.e., cystic echinococcosis is caused by *E. granulosus sensu stricto* and alveolar echinococcosis is caused by *E. multilocularis* [5]. Diagnosis is easily made in endemic areas from the history and radiologic investigations mostly, as in our case the CT scan gave us a high index of suspicion. Other tests include immunoelectrophoresis and enzyme-linked immunosorbent assay for diagnostic and screening purposes [6, 7]. The liver is the most commonly affected organ followed by the lungs, spleen, kidney, and brain. Mortality is not directly related to the disease but rather due to its complications. The disease progression is usually slow; hence, most patients remain asymptomatic [8].

In this case, lack of hepatic manifestation was a rare presentation. Intraoperative findings showed a cyst, 20 cm in diameter, white in color with clear fluid located in the right hemithorax at the lower lobe. Hydatidosis in our settings is usually treated medically at first with albendazole followed by surgery. Surgery was also done in two similar case presentations by Ghallab and Alsabahi and Anyfantakis et al. where surgical excision of the cyst has been recommended with good outcome in both cases [9, 10].

Smaller cysts are asymptomatic and are incidental findings in most cases [3]. Smaller cysts are medically managed in most circumstances, while complicated cysts need surgical intervention followed by albendazole or mebendazole administration [11]. There is no standard treatment for hydatid disease but options include medical pharmacotherapy, percutaneous drainage, and surgery. Generally, management is planned according to the World Health Organization diagnostic classification. Cysts less than 5 cm are treated with albendazole and those greater are managed by percutaneous drainage or surgery with or without albendazole [8]. Bulakçı et al. outline radical surgery to be the first-line treatment together with early diagnosis to optimize outcome. They also mention transplant surgery followed by immunosuppression in patients where surgical excision is not favorable. In addition, albendazole and mebendazole are the frequent parasitic drugs widely used [12].

## 4. Conclusion

Thoracic hydatid cysts are rare even in endemic areas. Diagnosis is based upon in-depth history, imaging, and histological analysis. Hydatid cysts have a good prognosis regardless of their size if removed completely without spillage. Hydatid disease remains to be of public health importance especially among this indigenous tribe (Maasai); therefore, a need of education and prevention is needed.
